# Estimation of hydrogen solubility in aqueous solutions using machine learning techniques for hydrogen storage in deep saline aquifers

**DOI:** 10.1038/s41598-024-76850-8

**Published:** 2024-10-29

**Authors:** Mohammad Rasool Dehghani, Hamed Nikravesh, Maryam Aghel, Moein Kafi, Yousef Kazemzadeh, Ali Ranjbar

**Affiliations:** 1 Department of Petroleum Engineering, Faculty of Petroleum, Gas, and Petrochemical Engineering, Persian Gulf University, Bushehr, Iran; 2 Department of Environmental Health Engineering, Faculty of Health and Nutrition, Bushehr University of Medical Sciences, Bushehr, Iran

**Keywords:** Underground hydrogen storage, Hydrogen solubility, Machine learning, Saline aquifers, Engineering, Chemical engineering

## Abstract

The porous underground structures have recently attracted researchers’ attention for hydrogen gas storage due to their high storage capacity. One of the challenges in storing hydrogen gas in aqueous solutions is estimating its solubility in water. In this study, after collecting experimental data from previous research and eliminating four outliers, nine machine learning methods were developed to estimate the solubility of hydrogen in water. To optimize the parameters used in model construction, a Bayesian optimization algorithm was employed. By examining error functions and plots, the LSBoost method with R² = 0.9997 and RMSE = 4.18E-03 was identified as the most accurate method. Additionally, artificial neural network, CatBoost, Extra trees, Gaussian process regression, bagged trees, regression trees, support vector machines, and linear regression methods had R² values of 0.9925, 0.9907, 0.9906, 0.9867, 0.9866, 0.9808, 0.9464, and 0.7682 and RMSE values of 2.13E-02, 2.43E-02, 2.44E-02, 2.83E-02, 2.85E-02, 3.40E-02, 5.68E-02, and 1.18E-01, respectively. Subsequently, residual error plots were generated, indicating the accurate performance of the LSBoost model across all ranges. The maximum residual error was − 0.0252, and only 4 data points were estimated with an error greater than ± 0.01. A kernel density estimation (KDE) plot for residual errors showed no specific bias in the models except for the linear regression model. To investigate the impact of temperature, pressure, and salinity parameters on the model outputs, the Pearson correlation coefficients for the LSBoost model were calculated, showing that pressure, temperature, and salinity had values of 0.8188, 0.1008, and − 0.5506, respectively, indicating that pressure had the strongest direct relationship, while salinity had an inverse relationship with hydrogen solubility. Considering the results of this research, the LSBoost method, alongside approaches like state equations, can be applied in real-world scenarios for underground hydrogen storage. The findings of this study can help in a better understanding of hydrogen solubility in aqueous solutions, aiding in the optimization of underground hydrogen storage systems.

## Introduction

**Figure 1:**
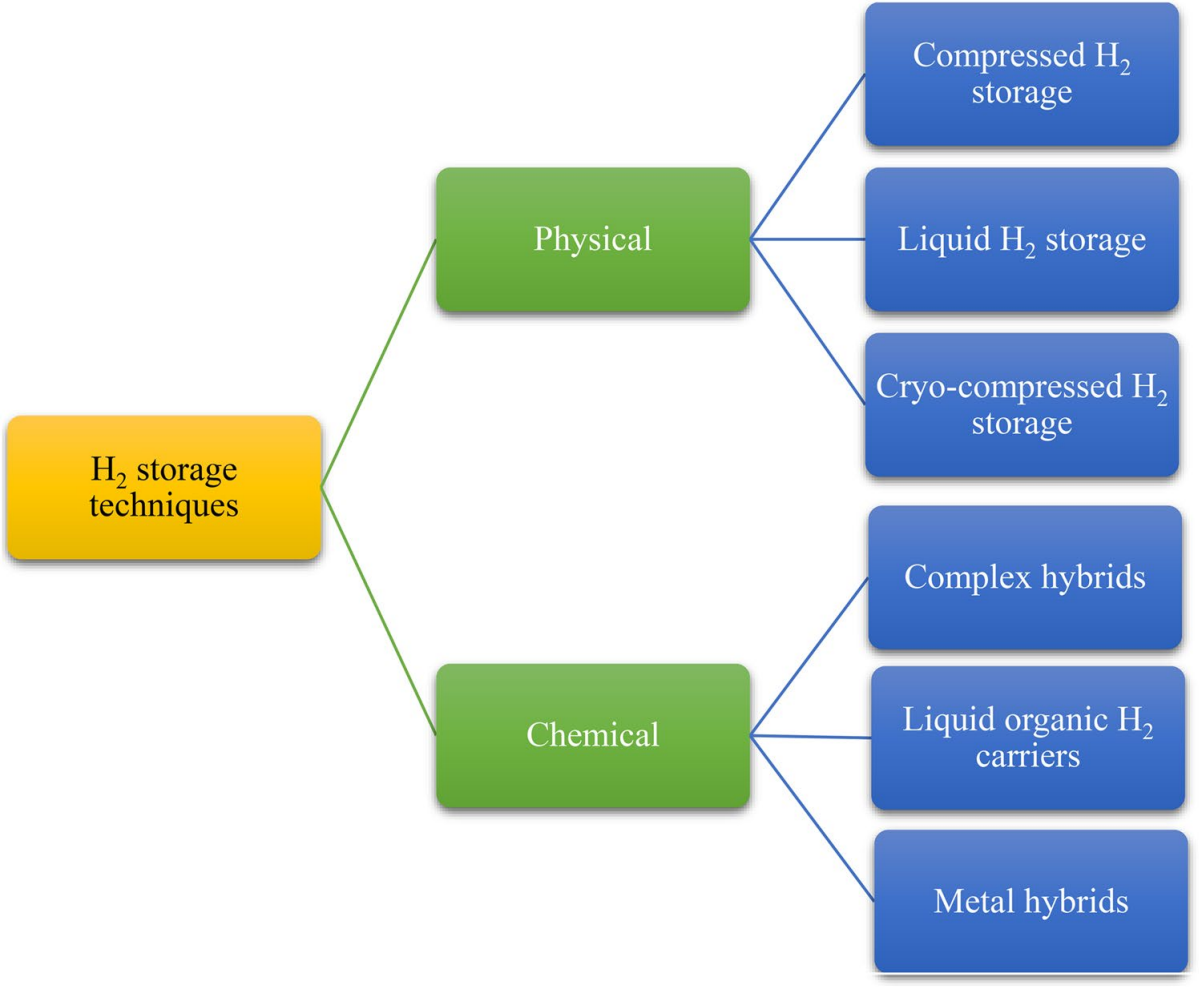
Hydrogen storage techniques^44^.

The widespread utilization of fossil fuels has prompted the emergence of environmental challenges, resource depletion, and health concerns^1^. Consequently, a critical response has been emphasized by researchers, focusing on sustainable and renewable energy alternatives to address these issues^2–5^. Minimizing or eliminating the use of fossil fuels, as well as developing green fuels with renewable aspects for current and future applications, is ultimately necessary^6^. Among the various clean energy sources, hydrogen has attracted attention due to its unique properties, including the highest energy content per unit mass and the production of only water during combustion^7–10^. Hydrogen can be used in various sectors, including transportation, power generation, and industry, and serves as a clean and efficient fuel^11–14^. Despite its potential as a clean fuel, widespread adoption of hydrogen faces significant challenges, particularly in storage and transportation^15–19^. Figure 1 illustrates hydrogen storage techniques. Storing hydrogen safely is complex due to its high chemical and microbial reactivity compared to other gases like natural gas or carbon dioxide, raising concerns about safety and environmental impact^16,20–22^. These properties of hydrogen increase the risk of leaks from storage environments and the loss of significant amounts of hydrogen, which requires careful management^23,24^. This brings to the forefront technical challenges such as selecting and designing appropriate geological storage environments, ensuring the integrity of wells and associated facilities, and managing the variable properties of hydrogen at underground conditions. Issues related to regular monitoring and maintenance to ensure safe and effective operation require innovative solutions and resistance to harsh geological conditions, which also depend on continuous research and development in this area^25,26^. One of the greatest advantages of hydrogen storage is the ability to store large amounts of energy for long periods^27–30^. This can be particularly useful when energy production from renewable sources such as solar and wind is intermittent^31–33^. Hydrogen storage can help balance energy supply and demand and prevent problems caused by fluctuations in energy production^34–36^. Additionally, using the subsurface for hydrogen storage can help reduce the environmental impacts associated with other energy storage methods^27,37^. This is because less valuable land can be used for this purpose, and there is no need to construct large, expensive above-ground structures^38,39^. However, underground hydrogen storage also has challenges and drawbacks. These include technical issues related to ensuring that hydrogen does not leak and maintaining its quality over time. Hydrogen leaks can pose risks such as fires and explosions and can also harm the environment^27,40,41^. Additionally, the initial costs of establishing underground hydrogen storage facilities can be very high. This includes the costs of drilling, constructing, and maintaining storage facilities, which may not be economically viable in some cases^30,42,43^.

Hydrogen is recognized as a clean fuel that produces only water vapor when burned or used in fuel cells. This property allows it to be used as an excellent option to reduce air pollution and combat climate change^45^. Hydrogen has a very high energy density, which means it can store large amounts of energy per unit mass. This property makes hydrogen ideal for use in vehicles and other applications that require large amounts of energy. Additionally, hydrogen can be produced through electrolysis of water using renewable energy sources, making hydrogen a sustainable energy source. On the other hand, hydrogen can act as a medium for energy storage and transmission, allowing renewable energy generated in different locations to be transported to other locations or times. Hydrogen also has applications in various industrial sectors such as steel production and electric power. Despite the challenges in infrastructure and storage, with the advancement of technology and the development of related infrastructure, these problems will gradually be solved and hydrogen can play a more important role in sustainable energy supply^1,6,45,46^.

There is a variety of methods for measuring hydrogen solubility in different mediums that can be found in the literature. Here, some of the most common methods are mentioned:

- Gas Chromatography: This method is a chemical analysis technique that uses a chromatography column to separate the components of a sample based on their physical and chemical properties^47,48^. When hydrogen from the sample moves through the column at a different speed than other substances present, the different components of the sample exit the column at different times^48^.
- Nuclear Magnetic Resonance (NMR) Spectroscopy: This method works based on the magnetic properties of atomic nuclei, especially hydrogen nuclei^49–51^. When the sample is placed in a strong magnetic field, the nuclei absorb radio pulses and then release energy as radio signals. These signals are analyzed to provide information about the type, number, and position of hydrogens in molecules^50,52^.
- Sieverts’ Method: This method is a common way to measure the solubility of gases (hydrogen) in metals. It uses the principles of physics and chemistry to determine the amount of hydrogen gas absorbed by metals. In this technique, the pressure of hydrogen gas in a chamber containing the metal sample is measured before and after hydrogen absorption by the sample^47^.
- Thermal Desorption Spectroscopy (TDS): In this method, the sample is heated to release hydrogen, which is then measured by specific sensors^53,54^.
- Machine Learning: This method uses data analysis and pattern recognition capabilities to estimate hydrogen solubility^55–58^. Machine learning models can be used to predict hydrogen solubility in different environments more accurately, which ultimately improves storage processes^55,59,60^. One of the most important applications of machine learning in this field is its use to estimate hydrogen solubility in deep saline aquifers^61,62^.

Several studies have investigated hydrogen solubility in different mediums using machine learning techniques. In 2022, Ansari et al. employed two machine learning approaches, Radial Basis Function, and Least Square Support Vector Machine, to estimate hydrogen solubility in aqueous solutions. In their study, the RBF method with the CA optimization algorithm was recognized as the most effective approach. This study has developed models in a very good and wide range of temperatures, pressures, and salinities. It has also compared the machine learning model with the equations of state. However, only two machine learning methods have been investigated in this study, and the focus is on optimization methods^63^. In 2022, Cao et al. focused on estimating hydrogen solubility using artificial neural networks along with three optimization methods. The highest accuracy in their study was attributed to the Radial Basis Function (RBF) algorithm. The strength of this study is the investigation of hydrogen solubility in various and wide-ranging temperatures, pressures, and salinities. However, the range of hydrogen solubility is relatively limited. Additionally, the variety of methods used is relatively low and all are ANN-based methods^64^. Furthermore, in 2024, Lv et al. investigated eight different Black-box and white-box machine learning methods. Among these, the AdaBoost-SVR method demonstrated the best performance in estimating hydrogen solubility. This study also examines a very wide range of inputs. Additionally, a significant advantage of this research is the presentation of various correlations for estimating hydrogen solubility. However, a comprehensive review and comparison of different black-box models still appears necessary^65^.

A crucial aspect overlooked in previous studies is the removal of outlier data before model building, which can significantly impact model accuracy and performance.

Table 1 summarizes previous studies on predicting hydrogen solubility using machine learning techniques.

**Table 1:** Previous studies on the prediction of hydrogen solubility in aqueous solutions using machine learning models.

Reference	Medium	Temperature (K)	Pressure (bar)	Salinity (mol/kg)	ML Models
Ansari et al.^63^	brine	273.15–636.1	1.01325-1013	0–5	RBF-BBO, RBF-CA, RBF-ICA, RBF-TLBO, LSSVM-BBO, LSSVM-CA, LSSVM-ICA, LSSVM-TLBO
Cao et al.^64^	brine	269.03–345.8	0.981–493.6	0–4	ANN-FBA, ANN-GA, ANN-RBF
Lv et al.^65^	brine	273-636.1	1.013–1013	0–5	AdaBoost-SVR, GB-SVR, BG-DT, AdaBoost-DT, KNN, GEP, GMDH, GP
Mohammadi et al.^66^	alcohols	213.15–524.9	1.01–1103	-	DeepESN, XGBoost, ELM, MARS
Zhou et al.^67^	alcohols	341.5–423	53-88.2	-	LSSVM, ANFIS2, ANFIS3, MLP, CFF, GR, RBF
Hadavimoghaddam et al.^68^	alcohols	213.15–524.9	1.01–1103	-	GP, GMDH
Cao et al.^69^	biomaterials	323.15–543.	38.9-265.56	-	MLP, CCNN, GRNN, LSSVR, GEP
Jiang et al.^60^	aromatic/cyclic compounds	298.15-575.15	0.001–620.1	-	MLPNN, CFFNN, RBFNN, GRNN, LSSVM, ANFIS
Mohammadi et al.^70^	hydrocarbons	298.15–691.6	2.2-301.5	-	XGBoost, MLP, AdaBoost-SVR, LiteMORT
Amar et al.^71^	hydrocarbons	92.3-583.45	1.01325-284.3	-	MLP, CFNN, CMIS

This study aims to estimate hydrogen solubility in deep saline aquifers using advanced machine learning techniques, which have revolutionized industries by automating tasks, analyzing vast datasets, and enhancing efficiency^72^. Machine learning is utilized to estimate hydrogen solubility in deep saline aquifers^61,62^, making it effective in reducing storage costs and increasing storage security and capacity^73^. With big data processing and evaluation algorithms, this process can be investigated more precisely, accurately predicting hydrogen solubility in different conditions^74,75^, and leading to the development of technologies successful in real conditions^76–78^. These techniques allow us to investigate hydrogen storage challenges innovatively, taking a significant step towards using this clean energy source effectively^79,80^.

This research investigates hydrogen solubility prediction in aqueous solutions using nine machine learning methods, including four advanced novel techniques: Gaussian Process Regression, Least-Square Boosting, Extra Trees, and Categorical Boosting. These methods were chosen for their capability to manage complex, nonlinear relationships and enhance prediction accuracy across diverse conditions. Unlike previous studies that often use a limited number of models, our work introduces a wider array of techniques, enabling a more robust approach to hydrogen solubility estimation. The findings of this study helps to create a comprehensive framework for tackling hydrogen solubility prediction challenges in deep saline aquifers, providing insights applicable to various real-world scenarios.

## Methodology

### Data collection and processing

In this study, to construct machine learning models, data were collected from previous researches available in various databases^81–87^. Table 2 shows data information gathered from each reference.

**Table 2:** Hydrogen solubility database used in this work.

Reference	Data range			
	Pressure (bar)	Temperature (K)	Salinity (%wt)	Solubility (mol fraction)
Wiebe and Gaddy 1934^86^	5.00-110.00	273.15-423.15	0.00–1.00	0.027–0.979
Morrison et al. 1952^82^	4.60-101.40	273.15-373.15	0.00–5.00	0.013–0.792
Ruetschi and Amlie 1966^87^	5.00-110.00	333.15-363.15	0.00	0.038–0.690
Crozier and Yamamoto 1974^81^	5.00–25.00	273.15-373.15	1.00–3.00	0.013–0.146
Gordon et al. 1977^85^	5.00–25.00	273.15-373.15	3.00–5.00	0.007–0.093
Kling and Maurer 1991^84^	5.00-110.00	273.15-423.15	0.00	0.002–0.913
Jáuregui-Haza et al. 2004^83^	11.64–196.60	323.20-423.15	2.50-5.00	0.000-0.004

All data were collected in a manner to covered a wide spectrum of temperature (K), pressure (bar), and salinity(%wt). This dataset consists of 230 data points and covers a pretty wide range of temperature, salinity, pressure, and solubility. Also, the database contains data for both pure and saline water.

**Figure 2:**
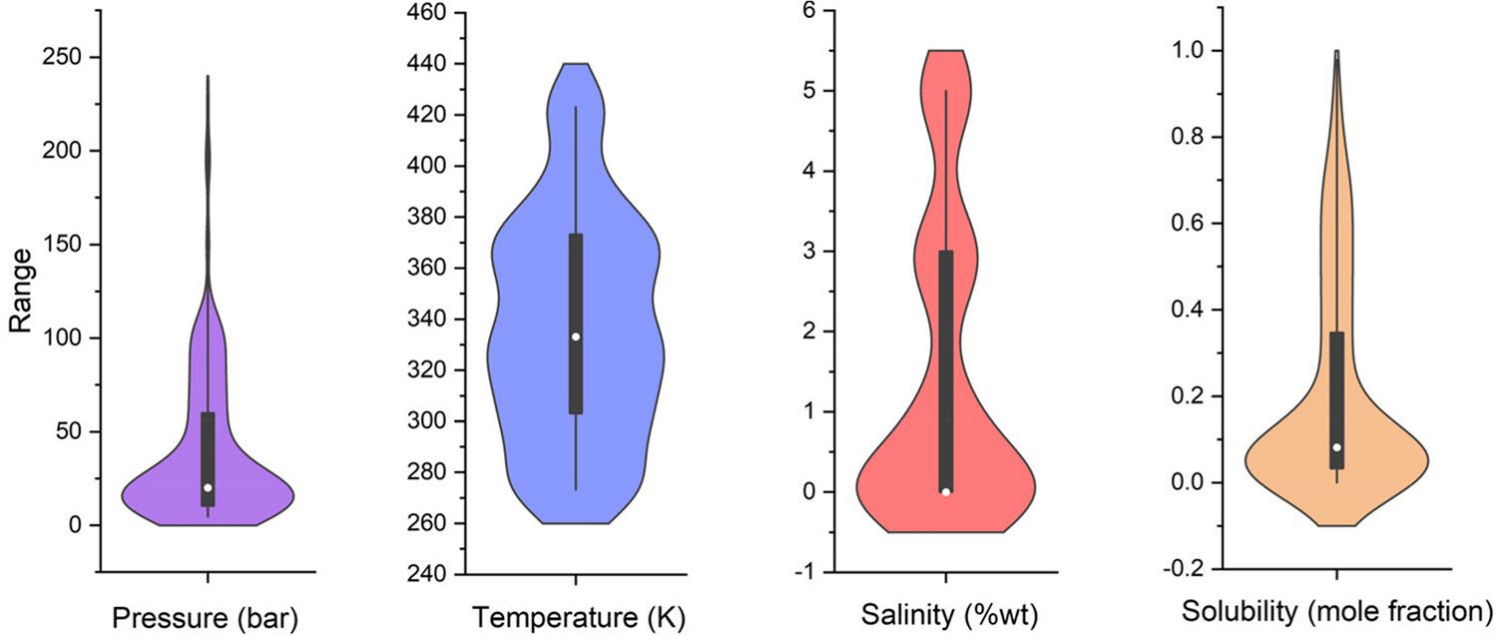
Violin with box plots of input and output parameters.

The violin with box plots of each input and output parameter is shown in Fig. 2.

To examine the impact of input parameters on each other and the output (hydrogen solubility), the Pearson linear correlation coefficient (PCC) was calculated for pairs of parameters by using Eq. (1). PCC measures the linear relationship between two variables or a set of variables, where the coefficient is computed as the ratio of the covariance of the variables to the product of their variances. A correlation value of + 1 indicates a perfect positive linear relationship between two variables, while a value of -1 indicates an inverse relationship. A value of 0 indicates no linear relationship^88,89^.

1 $$\:PCC=\frac{\sum(x_i-\overline x)(y_i-\overline y)}{\sqrt{\sum{(x_i-\overline x)}^2}\sqrt{\sum{(y_i-\overline y)}^2}}$$

In this equation, $\overline x$ and $\overline y$ represent the mean of parameters x and y, respectively.

**Figure 3:**
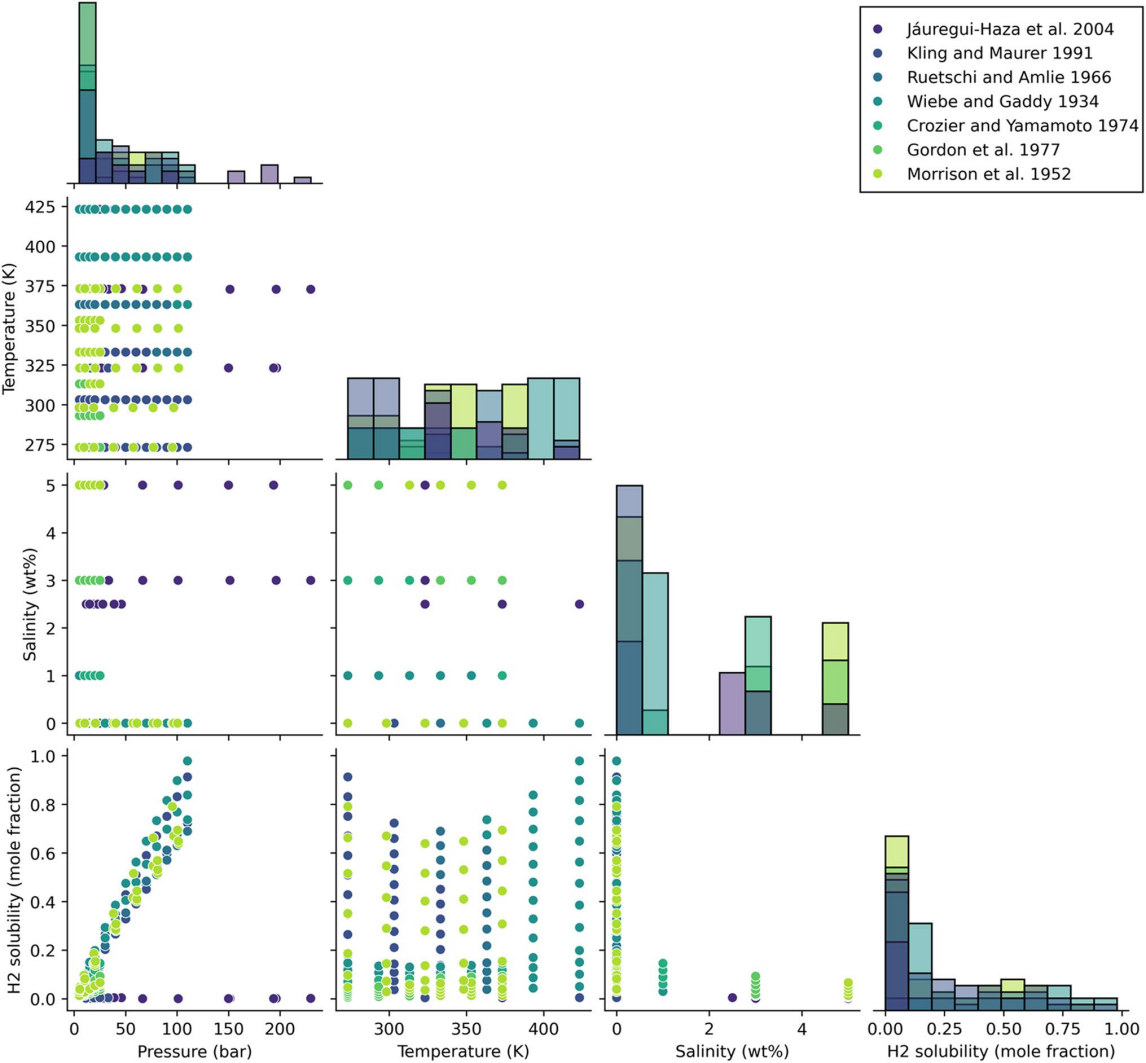
Scatter and histogram plots of parameters used to develop models.

**Figure 4:**
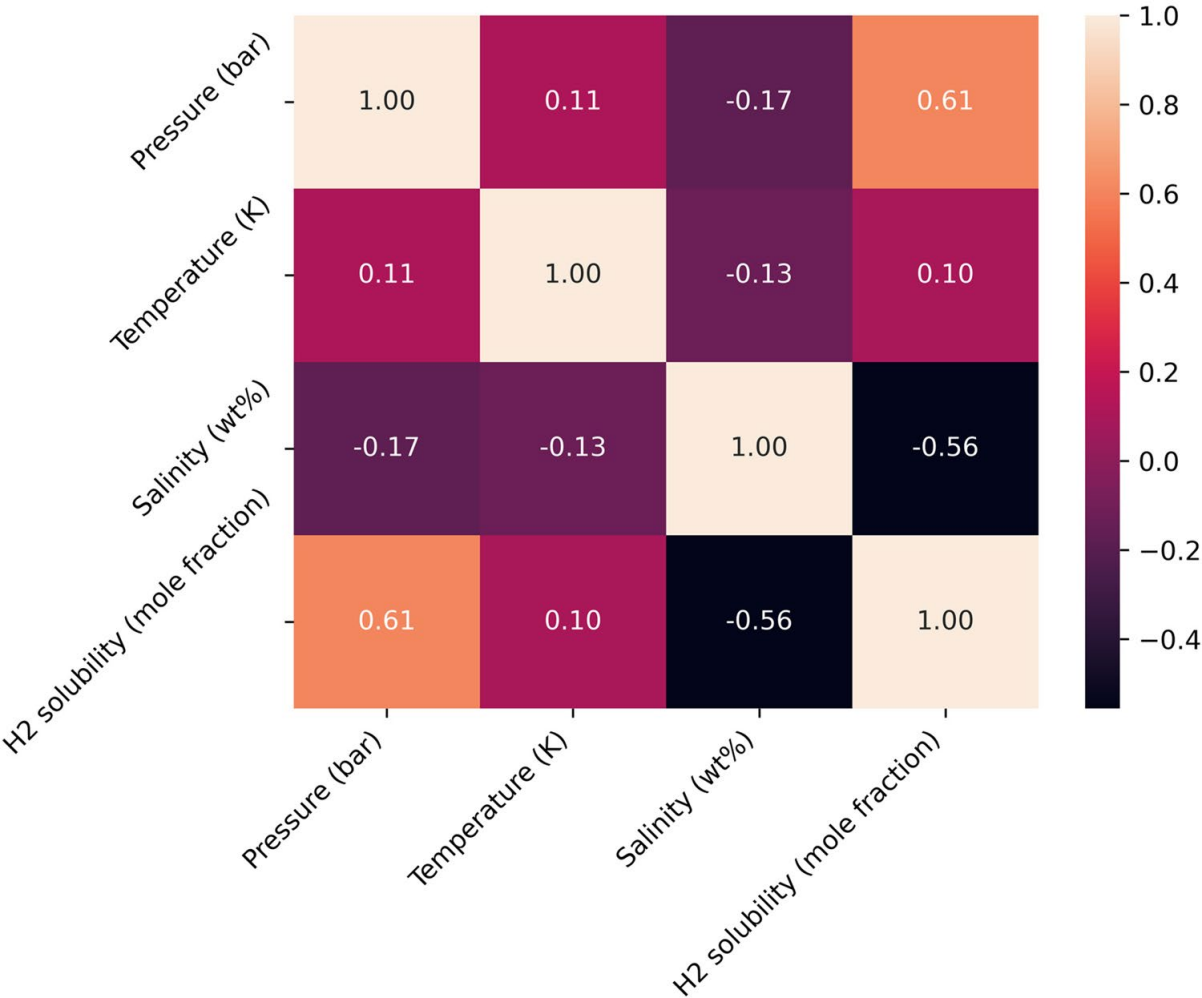
PCC values for each pair of data.

To assess and ensure the quality of the gathered data and correlation between parameters, scatter plots illustrating the parameters in relation to each other and histograms for each parameter and Pearson correlation coefficient for each pair of parameters are presented in Figs. 3 and 4. By examining Fig. 3, it can be seen that although the number of data related to pure water is more than the rest of the data, in general, the data has a favorable distribution. As evident from Fig. 4 among the input parameters, temperature exhibits the lowest correlation with hydrogen concentration, having a correlation coefficient of 0.0952. Additionally, among the other two parameters, pressure shows a positive correlation of 0.6070, while salinity demonstrates a negative correlation of 0.5563 with the output.

This study employed the standard deviation method to eliminate outlier data. Widely recognized in statistical analysis, this method entails computing the standard deviation of a dataset and subsequently excluding any data points that deviate beyond a specified number of standard deviations from the mean. The approach relies on two robust estimators, delineated as follows^90^:

2 $$\:2SD\:Method:\:\overline x\pm\:2SD$$

3 $$\:3SD\:Method:\overline x\pm\:3SD$$

Where $\overline x$ is the mean and SD is the standard deviation. Data that do not fall within these ranges are known as outlier data. In this study, the 3SD method was utilized.

In this case, only 4 data points were identified as outliers, and 226 data were used for models’ development, The statistical parameters for the remaining data points used in developing the machine learning models are presented in Table 3. Pre-processing techniques like normalization were omitted to mitigate potential issues like data leakage.

**Table 3:** Statistical data for input and output parameters.

Parameter	min	max	mean	Standard deviation	skewness	kurtosis	variance	range	median
Pressure(bar)	4.60	151.30	36.12	33.39	1.29	0.86	1114.59	146.70	20.00
Temperature(K)	273.15	423.15	335.98	44.09	0.28	-0.78	1944.06	150.00	333.15
Salinity(% weight)	0.00	5.00	1.40	1.86	0.91	-0.68	3.45	5.00	0.00
x_H2_(mole fraction)	0.0001	0.98	0.21	0.25	1.24	0.32	0.06	0.98	0.83

### Hyperparameter optimization

Hyperparameter optimization is required when using machine learning in engineering practice. Hyperparameters are parameters set in machine learning algorithms that are mainly used to control the performance of the model in terms of complexity, training speed, and generalization ability^91^.

Currently, hyperparameter optimization algorithms mainly include manual search, grid search, random search, and Bayesian optimization. Among them, manual search largely depends on the knowledge and experience of expert users, and parameter management becomes more difficult, especially as the range of parameters increases. Grid search is a comprehensive algorithm that achieves automatic parameter adjustment but is prone to the curse of dimensionality. To address the costly problems associated with grid search, random search is proposed. This method has higher search efficiency but also has some unreliability for complex models^92^.

In this study, the Bayesian algorithm (BS) was utilized to optimize the hyperparameter of base models: linear, artificial neural network, regression tree, support vector regression, bagged trees, Least-square boosting, extra trees, categorical boosting, and Gaussian process regression. Bayesian optimization is an adaptive hyperparameter search method, which builds an agent model based on Gaussian process and can predict the next combination that may bring the greatest benefit according to the currently tested hyperparameter combinations. Compared with other algorithms, it can make full use of historical information and find the optimal hyperparameters with fewer iterations^92^.

The BS algorithm operates based on Bayes’ theorem, which provides a statistical framework for updating probabilities in light of new information. This algorithm is particularly useful in machine learning for classification tasks. It starts with prior probabilities and updates them to calculate posterior probabilities as new data is received. Using observed data, Bayesian methods build accurate predictive models that are robust to data noise^93,94^.

One of the key advantages of the Bayesian algorithm is its ability to manage uncertainty and produce probabilistic predictions. Due to its simplicity and high efficiency, it is used in various applications, such as spam filtering, disease diagnosis, and large-scale data analysis. This makes it a critical tool in both classification tasks and the analysis of complex data^94,95^.

The mathematical formula of the BS algorithm is based on Bayes’ theorem and is expressed as follows:

4 $$\:P\left(H\right|E)=\frac{P\left(E|H\right).P\left(H\right)}{P\left(E\right)}$$

In this formula:

- (P(H|E)) is the posterior probability of a hypothesis (H) given evidence (E),
- (P(E|H)) is the likelihood, the probability of observing (E) assuming (H) is true,
- (P(H)) is the prior probability of (H),
- (P(E)) is the marginal likelihood, the overall probability of the evidence (E).

**Figure 5:**
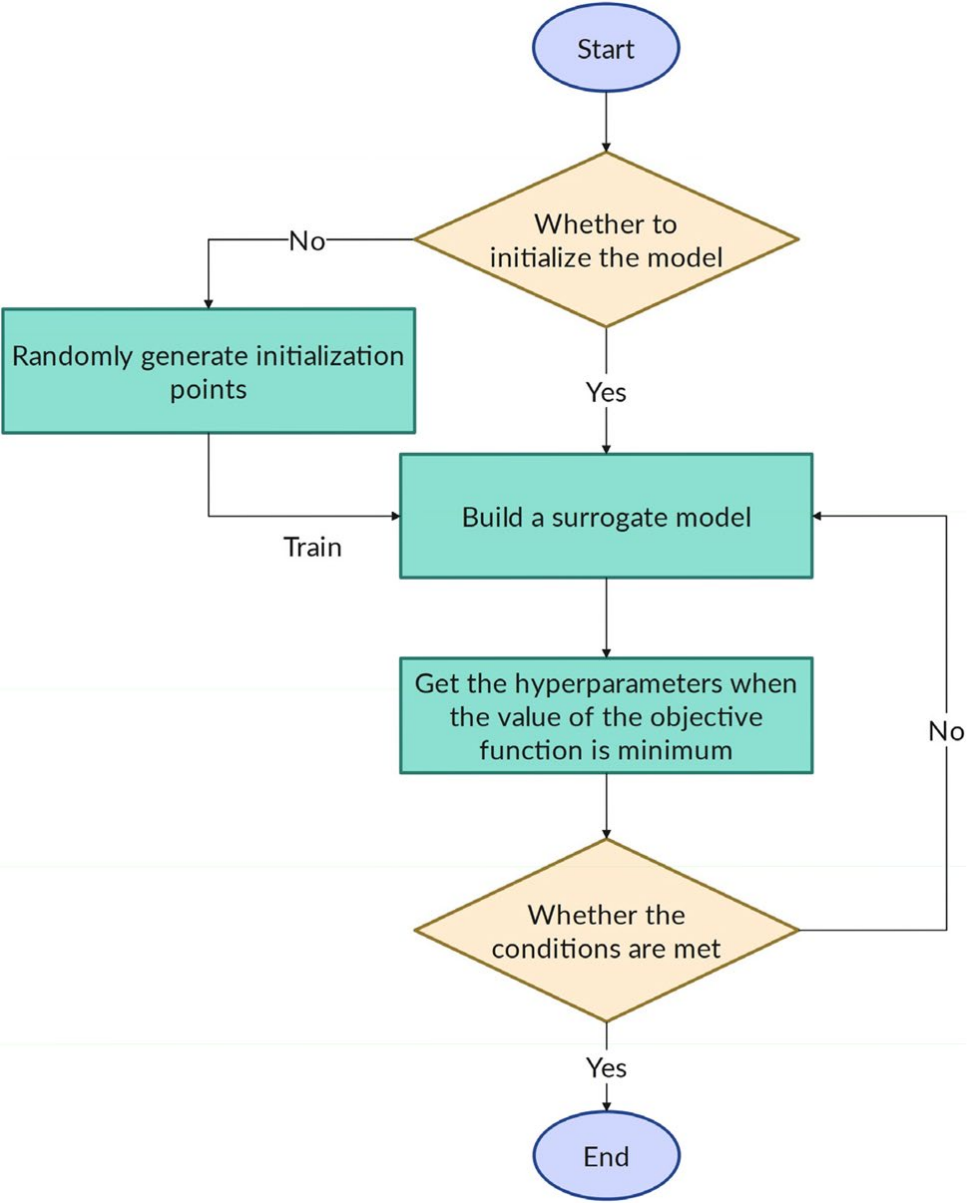
Schematic of Bayesian optimization^97^.

This formula helps update the probability of hypotheses based on new data^96^. A schematic of this algorithm is illustrated in Fig. 5.

### Development of machine learning models

After collecting and removing outliers from the data, nine regression methods were developed to predict hydrogen solubility. Due to the relatively small number of data, Initially, 90% of the data were randomly selected as the training set to ensure that the models can understand the relationships and complexity of the data, and the remaining 10% as the testing set to assess the models’ performance. Subsequently, Bayesian optimization with 30 iterations and 5-fold cross-validation was employed for the models to optimize their parameters and prevent overfitting.

#### Multiple linear regression

Linear regression is a statistical method used to investigate the relationship between a dependent variable and one or more independent variables which was developed by Galton for the first time in 1886^98^. The primary objective is to predict the dependent variable based on the independent variables. In linear regression, the hypothesis is that the relationship between the variables is approximable by a straight line. In other words, although the data may exhibit fluctuations, their overall relationship can be represented by a simple linear model^99,100^. Creating a linear regression model involves utilizing a dataset of observed values. Subsequently, the parameters of the regression line are adjusted using the method of least squares, aiming to minimize the sum of squared distances between data points and the prediction line. The primary advantage of linear regression is its ease of interpretability. As lines connecting the data points are easily understandable, examining the effects of independent variables on the dependent variable becomes more straightforward^101,102^.

Simple regression refers to regression analyses that link a single dependent or predicted variable to an independent or predictor unit. In such cases, linear regression analysis aligns with straight lines as depicted in Eq. (5) and Eq. (6)^103^:

5 $$\mathrm{Simple}\;\mathrm{Linear}\;\mathrm{regression}:\;\mathrm{Output}\;=\;C_0+C_1X+\varepsilon$$

6 $$\:\text{M}\text{u}\text{l}\text{t}\text{i}\text{p}\text{l}\text{e}\:\text{L}\text{i}\text{n}\text{e}\text{a}\text{r}\:\text{r}\text{e}\text{g}\text{r}\text{e}\text{s}\text{s}\text{i}\text{o}\text{n}\:\left(\text{M}\text{L}\text{R}\right):\:\:\text{O}\text{u}\text{t}\text{p}\text{u}\text{t}\:={C}_{0}+{C}_{1}{X}_{1}+{C}_{2}{X}_{2}+\dots\:+{C}_{n}{X}_{n}+\epsilon\:$$

In the above equations, “output” represents the estimated parameter, C_0_ is the intercept of the linear regression, and C_1_ to C_n_ indicate the influence of independent variables X_1_ to X_n_ on the output. ε denotes the difference between actual values and predicted values^103^.

Since linear regression has no hyperparameter, Bayesian algorithm was employed to minimize sum of squared error and determine the coefficients of the model. The final form of MLR optimized with the Bayesian algorithm (MLR-BS) can be stated as follows:

7 $$\:{x}_{H2}\:=0.1028+0.0053*Pressure-4.564*{10}^{-5}*Temperature-0.0457*Salinity+\epsilon\:$$

#### Artificial neural networks

The concept of neural networks dates back to the 1940s. The foundational work was by McCulloch et al., who proposed a simplified model of a neuron and introduced the idea of a neural network^104^. Artificial Neural Networks (ANNs) are computational structures inspired by the mechanisms of the human brain. These networks consist of a large number of computational units called neurons that are interconnected and used for solving problems such as pattern recognition, prediction, and control. Each neuron receives inputs, multiplies them by specific weights, and then sums them up^105^. Then, it transforms this sum through an activation function, which depends on the neuron’s active or inactive state. ANNs typically consist of multiple layers of neurons, including the input layer, hidden layers, and output layer. Each layer comprises several neurons that receive information from the previous layer and transmit it to the next layer. Learning in ANNs is achieved through optimization algorithms such as gradient descent. These algorithms enable the network to adjust neuron weights in a way that produces the desired output for different inputs^106,107^.

A typical model of a neuron includes a set of synapses, each characterized by its specific weight or strength. Other elements that constitute a neuron are: summation, activation function, and a bias. Mathematically, a neuron k can be described by the Eq. (8):

8 $$\:{U}_{k}=\sum\limits_{j=1}^{n}{x}_{j}{w}_{k,j}$$

In this equation, w and x represent the weights of the ANN model and feature, respectively^108^.

**Figure 6:**
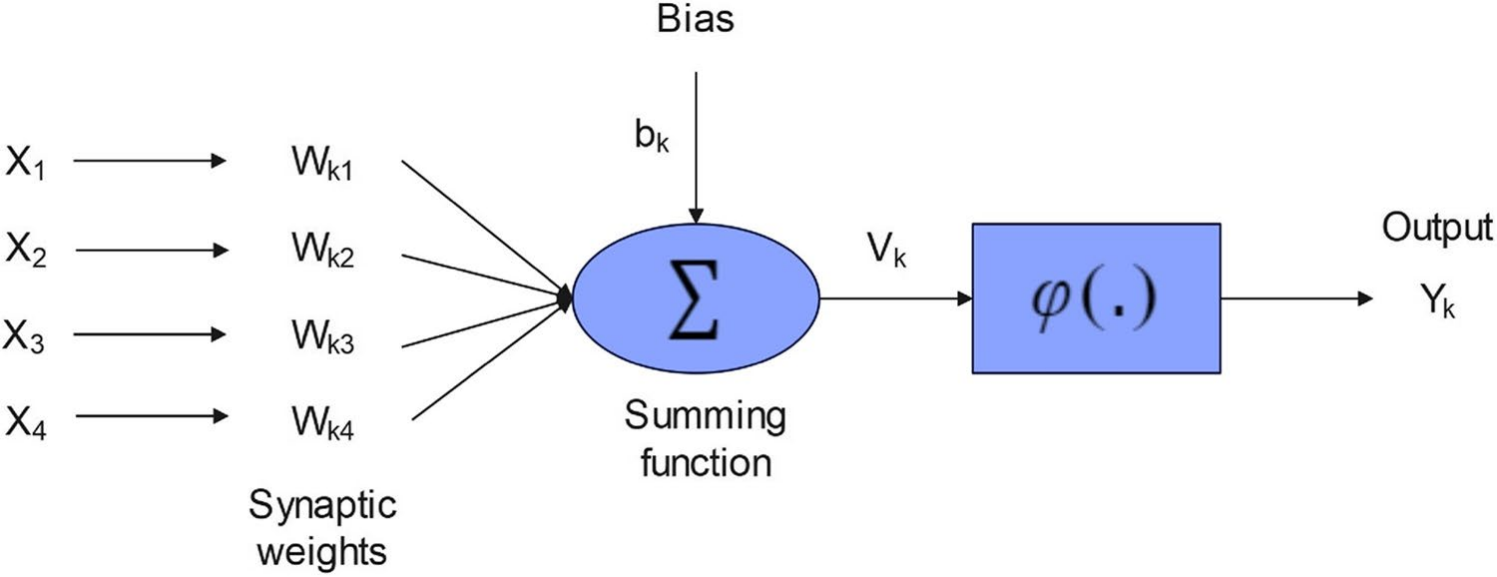
Model of an ANN^108^.

A schematic of an ANN is shown in Fig. 6.

The hyperparameters of the ANN model optimized with the Bayesian algorithm (ANN-BS) are shown in Table 4.

**Table 4:** Parameters of developed ANN-BS model.

Hyperparameter	Value
Hidden layers	5
Neurons in each layer	10
Training function	Levenberg-Marquardt

#### Regression tree

**Figure 7:**
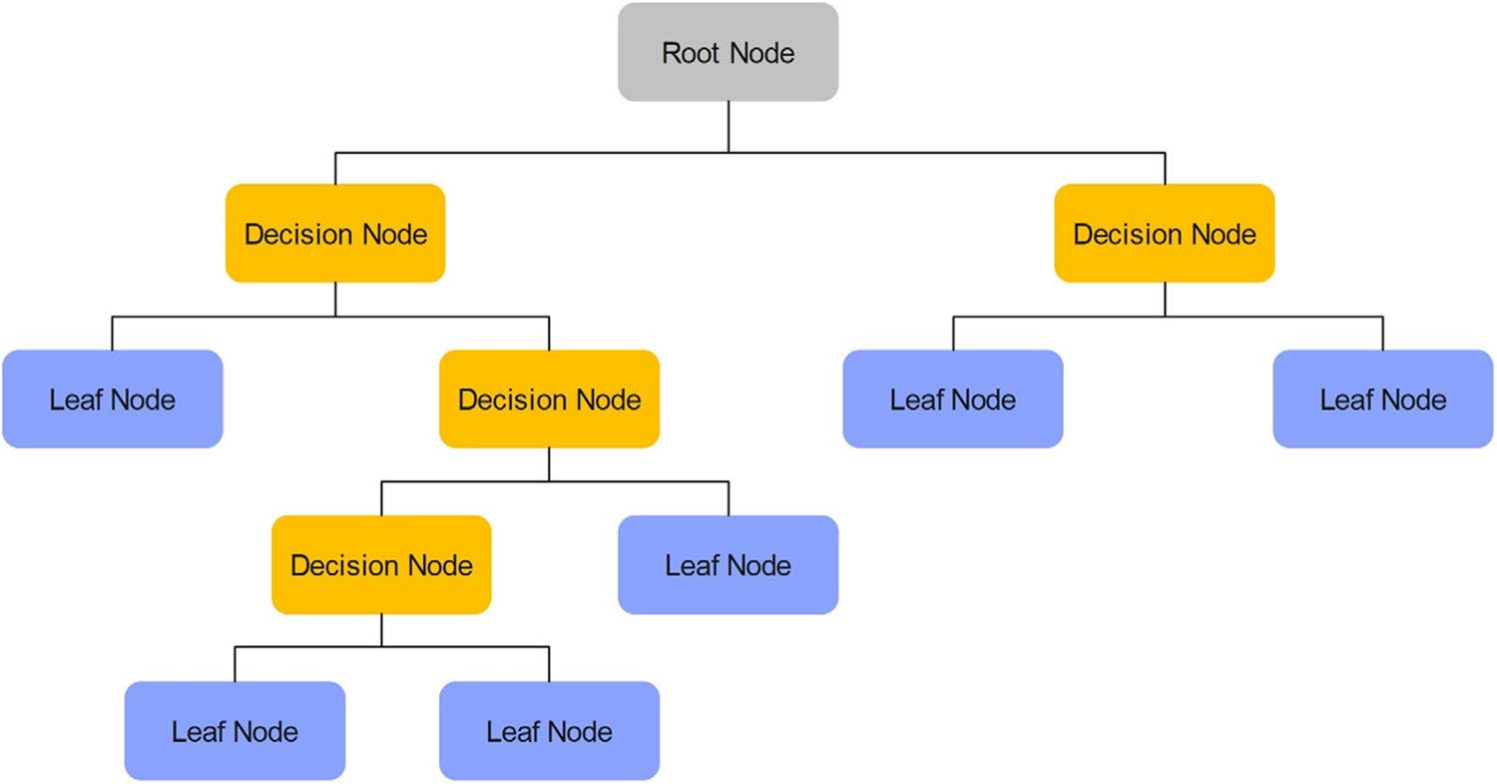
Schematic of a RT^113^.

Classification and regression tree methodology was introduced in 1984 by Breinman et al.^109^ Regression trees (RTs) are intuitive, practical, and accurate forecasting techniques and have been used in many fields. Apart from their predictive capabilities, regression trees can also cluster a data set. Trees recursively partition the dataset and find more homogeneous groups at each step. The tree is driven by predictability and the result can be interpreted as a clustering solution. This makes RTs a visual clustering algorithm, where clusters should have a predictable mean^110^. RTs can perform two types of cluster analysis: constrained clustering and unconstrained clustering. Constrained clustering, often referred to as profiling, aims to identify groups within a dataset that also exist in another. RTs are well-suited for profiling a dataset. The tree divides the dataset into nodes based on the value of a descriptive variable. The goal is to predict the values of the response variable for each observational unit, which is left down the tree through terminal nodes^111,112^. The tree attempts to minimize the sum of squared individual observations within the terminal nodes regarding their average, forming clusters in the response space. Thus, RTs characterize a response dataset into a descriptive dataset. The way a decision tree operates is illustrated in Fig. 7^113^.

The minimum leaf size for the RT model optimized with the Bayesian algorithm (RT-BS) has been calculated to be 1.

#### Support vector regression

Support Vector Regression (SVR) is a modeling method used to predict continuous values (similar to linear regression) which was first introduced by Drucker et al. in 1996^114^. However, instead of using regular lines or curves to fit the data, it employs the concept of support vectors. In SVR, similar to the classical Support vector machines for classification, it aims to determine an optimal margin between the available data and the regression line or surface. This margin is created by support vectors, which are the data points farthest from the training data. Like the classical method, SVR also utilizes Kernel Functions to model more complex patterns. The advantages of SVR include its robustness against outliers and noisy data, and its ability to handle large datasets. Additionally, it excels at modeling nonlinear relationships. However, it has limitations, such as high computational resource consumption and the need for careful configuration of hyperparameters. These challenges may pose difficulties in the effective use of Support Vector Regression^115,116^. SVR cost function can be defined as follows:

9 $$\:{\left|y-f\left(x\right)\right|}_{\epsilon\:}=\left\{\begin{array}{c}\:\:0\:\:\:\:\:\:\:\:\:\:\:\:\:\:\:\:\:\:\:if\:\:\:\left|y-f\left(x\right)\le\:\epsilon\:\right|\\\:\left|y-f\left(x\right)\right|-\epsilon\:\:\:\:\:\:\:\:\:\:\:\:\:\:\:\:\:\:\:\:\:\:\:\:\:\:\:\:\:\end{array}\right.$$

In Support vector regression, we aim to find an approximating function f(x) for the dependent variable y. In the given formula, $\:{\left|y-f\left(x\right)\right|}_{\epsilon\:}$ represents the distance between the actual value y and the predicted value f(x), determined by the parameter ε. If this distance is less than or equal to ε, it is assumed that the model has made an acceptable prediction, and the error is zero ($\:{\left|y-f\left(x\right)\right|}_{\epsilon\:}=0$). However, if this distance is greater than ε, we are faced with $\:\left|y-f\left(x\right)\right|-\epsilon\:$, indicating the model error. In this case, we attempt to minimize this error to provide a more accurate model. In the terminology of Support vector regression, we aim to minimize this error by considering margin-related constraints, thus presenting a regression model with precision and suitable performance^116^.

The hyperparameters of the optimized SVR model with bayesian (SVR-BS) are given in Table 5.

**Table 5:** Developed SVR-BS model hyperparameters.

Hyperparameter	Value
Box constraint	107.119
Kernel function	Gaussian
Kernel scale	13.2163
Epsilon	0.002415
Standardize data	True

#### Gaussian process regression

The foundational theory of Gaussian Processes can be traced back to the time-series analysis work of Wiener and Kolmogorov^117^. Gaussian Process Regression (GPR) is a method for modeling nonlinear relationships between dependent and independent variables. This approach is based on the assumption that the distribution of each data point follows a Gaussian (normal) process. In this method, a Gaussian random variable is assigned to each data point. Then, using the information available in the training data points, a Gaussian distribution is estimated for the entire input space. This Gaussian distribution represents the probability distribution of possible values for the dependent variable at each point in the input space^113,118^. One interesting feature of GPR is that, instead of adjusting model parameters (as in linear modeling methods), the model tunes its parameters based on the data by using the data itself as the centers of Gaussian distributions. In other words, GPR utilizes the data as centers of Gaussian distributions for the dependent variable. To predict new values, the Gaussian distribution derived from the training data is updated based on the new data. The flexibility of GPR in modeling complex relationships and its ability to generalize to nonlinear problems are some of its advantages. However, this method requires computationally expensive operations for analyzing large datasets and may also need parameter tuning^119^.

The hyperparameters of the GPR model optimized with Bayesian algorithm (GPR-BS), are listed in Table 6.

**Table 6:** Developed GPR-RS model hyperparameters.

Hyperparameter	Value
Sigma	0.0091398
Basis function	Zero
Kernel function	Isotropic squared exponential
Kernel scale	0.92534
Standardize	True

#### Bagged trees

Bagged Tree models are an effective and powerful method for modeling and prediction which was introduced by Breiman for the first time^120^. This approach involves combining multiple decision trees to increase the model’s accuracy and performance. The concept of Bagged Trees is to create several independent decision trees. Each decision tree is constructed using a subset of the training data, employing methods such as Bootstrap Sampling. This method allows each tree to build a different model with different training data. When making predictions, all decision trees are executed on the input data, and the prediction results of each tree are combined^121^. This combination is usually performed by averaging in the model (Bagging). The advantages of Bagged Trees include increased robustness, reduction of bias and variance, and improved performance in predicting new data. This model is typically more stable than a regular decision tree and can handle outliers or noisy data better. On the other hand, Bagged Trees may require a high computational cost as multiple decision trees need to be trained and executed. Additionally, this model may be prone to overfitting if the number of trees is excessive or if proper settings are not applied^122^.

hyperparameters of the optimized BT model with the Bayesian algorithm (BT-BS) are shown in Table 7.

**Table 7:** Developed BT-BS model hyperparameters.

Hyperparameter	Value
Number of learners	29
Minimum leaf size	1
Learn rate	1

#### Least-square boosting

Least-square boosting (LSBoost) is a powerful modeling method used for prediction and classification problems which was first introduced by Friedman^123^. This approach combines multiple decision trees, often referred to as “weak models,” to create a “strong model” for prediction. The Boosting process starts by creating a decision tree that fits well with the training data. Then, at each stage, the focus is on data points that have been misclassified. A new model (typically another decision tree) is created to correct these errors. One key feature of LSBoost is that at each stage, the new model is selected in a way that minimizes the errors from the previous stages^124^. This process continues until the errors decrease to an acceptable level or a specified number of stages is completed. One popular type of LSBoost model is Gradient Boosting, where, at each stage, the model strives to minimize a cost function (typically differentiable). This method allows the model to gradually achieve the best predictions^125^.

Table 8 represents the hyperparameters of the LSBoost model optimized with the Bayesian algorithm (LSBoost-BS).

**Table 8:** Developed LSBoost-BS model hyperparameters.

Hyperparameter	Value
Number of learners	189
Minimum leaf size	1
Learn rate	0.1

#### Extra trees

Extra Trees was introduced by Geurts et al. in 2006^126^. The theoretical foundations of the Extra Trees (ET) method are based on a combination of the principles of RTs and Random Forests (RF). This method is a machine learning model that uses a “tree ensemble” approach, leveraging randomness in feature selection and split points to build the trees.

In decision tree methods, data is recursively split into different nodes to create predictive models. The final model is built using several randomized trees, each independently generated from the training data. One key difference between ET and RF is that ET introduces more randomness in the selection of features and split points, which leads to reduced model variance and helps prevent overfitting^126^.

**Figure 8:**
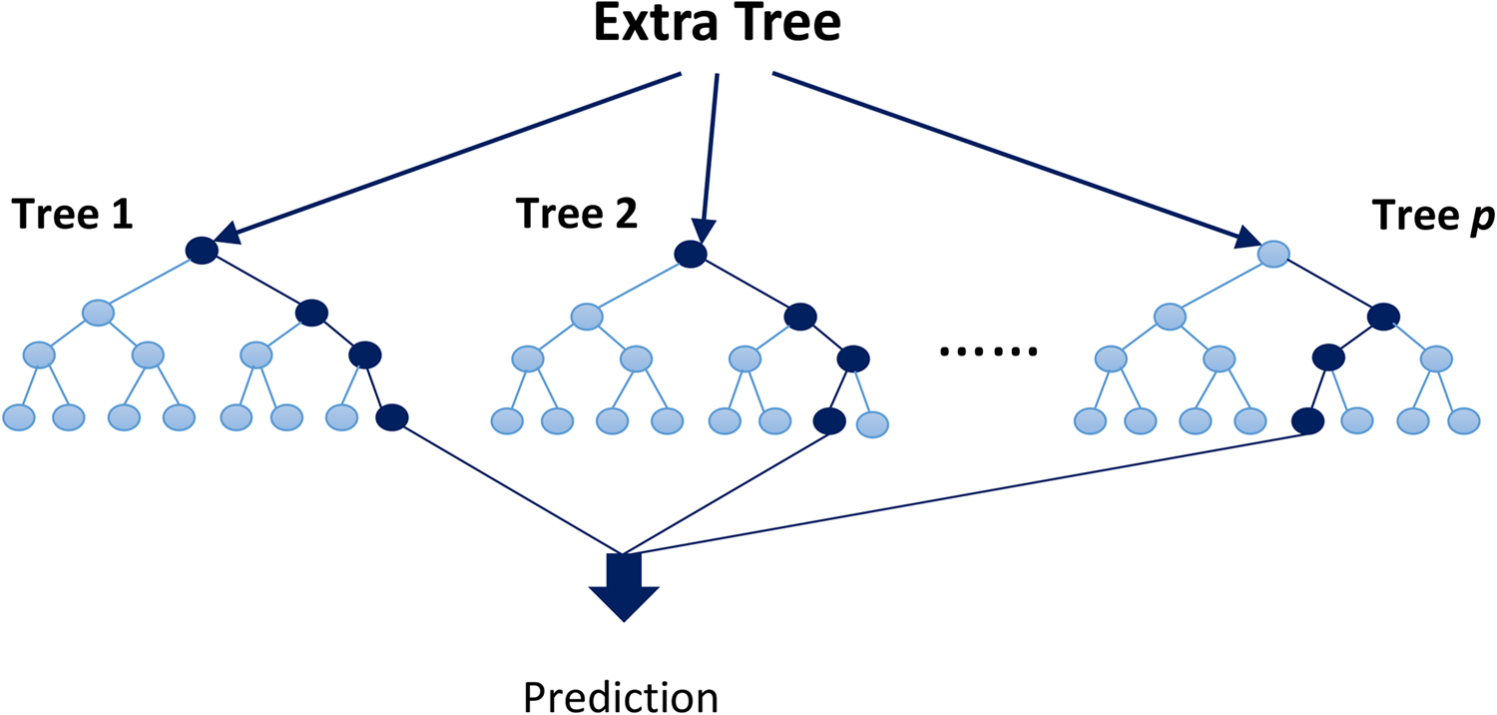
Illustration of extra trees model^128^.

Theoretically, this method is based on information theory, aiming to reduce generalization error through randomization. Unlike RF, ET does not optimize tree structure based on labeled data; instead, the tree structure is fully randomized, reducing the model’s error. Additionally, this method offers advantages such as high computational efficiency and the ability to make faster predictions^127^. ETs are defined using an unlimited number of random trees. Figure 8 shows an illustration of the ET method hyperparameters of the optimized ET model with the Bayesian algorithm (ET-BS) are shown in Table 9.

**Table 9:** Developed ET-BS model hyperparameters.

Hyperparameter	Value
Number of learners	237
Minimum sample split	7
Max depth	18
Minimum leaf size	3
Random state	101

#### Categorical boosting

Categorical Boosting (CatBoost) was developed by Prokhorenkova et al. and first introduced in 2018^129^. CatBoost is a machine learning library specifically designed for classification and prediction problems involving both textual and numerical data. Developed by Yandex, a leading Russian tech company, it utilizes Gradient Boosting algorithms for modeling^129^. One standout feature of CatBoost is its ability to automatically handle textual data without requiring complex preprocessing. This library is particularly well-suited for dealing with categorical data, efficiently leveraging the features of such data types. CatBoost also improves prediction accuracy through advanced techniques like Ordered Boosting and dynamic combinations during model learning^130^. Unlike other similar algorithms such as XGBoost and LightGBM, CatBoost effectively manages issues related to overfitting while delivering higher performance across various tasks. It is user-friendly, requires minimal configuration, and can be directly applied to datasets, making it a popular tool in data mining and analysis projects^131^. CatBoost employs Gradient Boosting algorithms for modeling, with its mathematical formulations similar to other Gradient Boosting algorithms but enhanced to handle categorical data. It is generally defined by the following equation:

10 $$\:F\left(x\right)=\sum\limits_{m=1}^{M}{\gamma\:}_{m}{h}_{m}\left(x\right)$$

Where F(x) is the final predictive model, γ_m_ is the model weights, and h_m_(x) represents the decision trees^123^.

Table 10 represents the hyperparameters of the CatBoost model optimized with the Bayesian algorithm (CatBoost-BS).

**Table 10:** Developed CatBoost-BS model hyperparameters.

Hyperparameter	Value
Iterations	146
Depth	8
Learn rate	0.073
Bagging temperature	0.85
Border count	201
Leaf estimation iterations	7
Random strength	0.45
Subsamples	0.78

## Results and discussion

### Performance of the developed models

#### Error factors

After constructing and optimizing the models, to assess their performance on training and test data, values for several error measurement methods, including mean absolute error (MAE), mean square error (MSE), root mean square error (RMSE), and coefficient of determination (R^2^), were calculated for both datasets. A value closer to one for R^2^ signifies better model performance and lower values for the other three methods indicate greater success of the model. The formulas for calculating these methods are provided below.

11 $$\:MAE=\frac{1}{N}\sum\limits_{i=1}^{N}\left|{output}_{i,exp}-{output}_{i,pred}\right|$$

12 $$\:MSE=\frac{1}{N}\sum\limits_{i=1}^{N}{\left({output}_{i,exp}-{output}_{i,pred}\right)}^{2}$$

13 $$\:RMSE=\sqrt{\frac{1}{N}\sum\limits_{i=1}^{N}{\left({output}_{i,exp}-{output}_{i,pred}\right)}^{2}}$$

14 $$\:R^2=1-\frac{\sum_{i=1}^N\left({output}_{i,exp}-{output}_{i,pred}\right)^2}{\sum_{i=1}^N\left({output}_{i,exp}-\overline{output_{exp}}\right)^2}$$

In the above formulas, $\:{output}_{exp}$, $\:{output}_{pred}$, $\overline{output_{exp}}$, and N represent the actual values of the output parameter, predicted values of the output parameter, the mean of actual output values, and the number of samples, respectively^65,132–135^.

**Figure 9:**
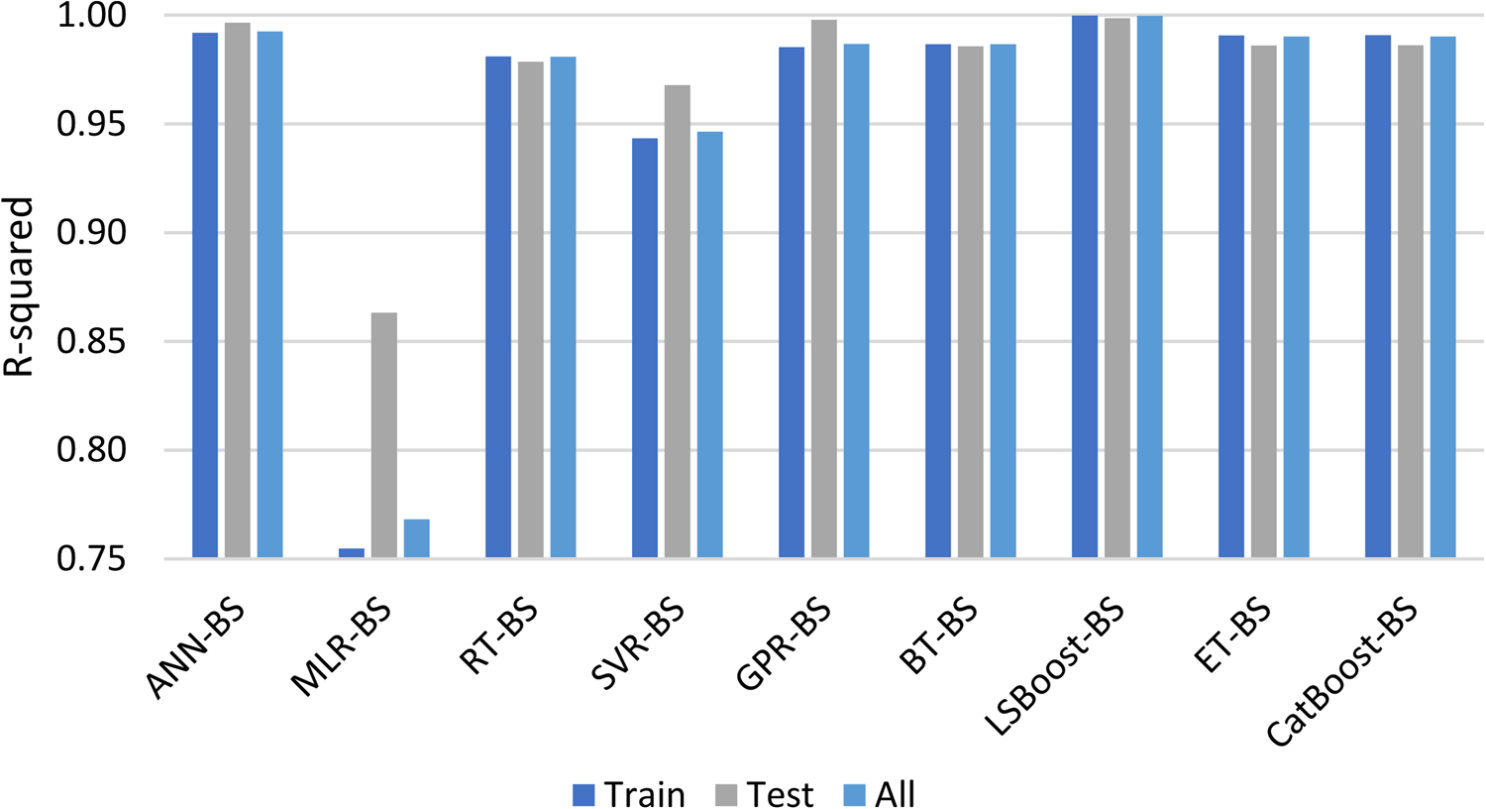
Illustration of R-squared for developed models.

Table 11 shows the performance of the developed methods on training, testing, and all data sets. Also, Fig. 9 illustrates R^2^ values for each model.

**Table 11:** Error factors of developed models.

	*R*^2^			MAE			RMSE			MSE		
	Train	Test	Total	Train	Test	Total	Train	Test	Total	Train	Test	Total
ANN-BS	0.9919	0.9965	0.9925	1.04E-02	1.19E-02	1.05E-02	2.19E-02	1.54E-02	2.13E-02	4.78E-04	2.36E-04	4.54E-04
MLR-BS	0.7548	0.8632	0.7682	7.48E-02	6.91E-02	7.42E-02	1.20E-01	9.64E-02	1.18E-01	1.45E-02	9.29E-03	1.40E-02
RT-BS	0.9810	0.9785	0.9808	1.69E-02	2.74E-02	1.80E-02	3.35E-02	3.82E-02	3.40E-02	1.12E-03	1.46E-03	1.16E-03
SVR-BS	0.9434	0.9678	0.9464	2.82E-02	2.35E-02	2.77E-02	5.79E-02	4.67E-02	5.68E-02	3.35E-03	2.19E-03	3.23E-03
GPR-BS	0.9852	0.9978	0.9867	1.62E-02	1.06E-02	1.56E-02	2.95E-02	1.24E-02	2.83E-02	8.73E-04	1.53E-04	8.00E-04
BT-BS	0.9866	0.9856	0.9866	1.57E-02	2.19E-02	1.64E-02	2.81E-02	3.12E-02	2.85E-02	7.91E-04	9.76E-04	8.10E-04
LSBoost-BS	0.9998	0.9986	0.9997	2.31E-03	7.20E-03	2.80E-03	3.00E-03	9.59E-03	4.18E-03	9.02E-06	9.20E-05	1.75E-05
ET-BS	0.9906	0.9860	0.9901	8.56E-03	2.05E-02	9.78E-03	2.36E-02	3.08E-02	2.44E-02	5.57E-04	9.51E-04	5.97E-04
CatBoost-BS	0.9907	0.9861	0.9902	1.54E-02	2.13E-02	1.60E-02	2.35E-02	3.07E-02	2.43E-02	5.52E-04	9.42E-04	5.92E-04

To assess the possibility of overfitting in the model, the error values of test and training data were compared across different methods. Since the error values did not show significant differences among the methods, it can be concluded that the models did not experience this phenomenon.

#### Graphical visualization

**Figure 10:**
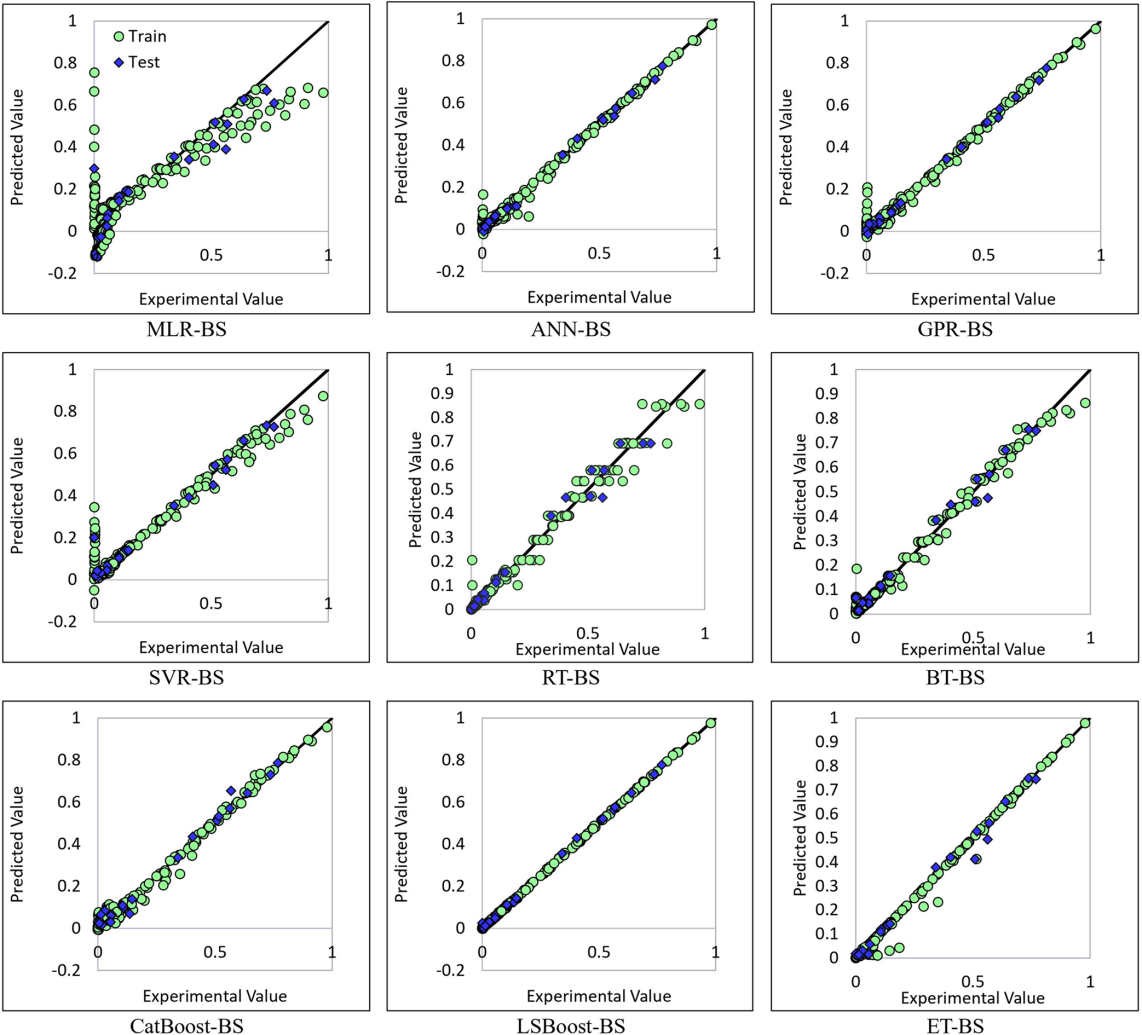
Cross plots for developed models.

Another method for assessing the success of a model in predicting the output parameter is to plot the predicted data points against the actual values. It is evident that the more points on this plot align with the X = Y line, the better the model’s performance in predicting the output. Figure 10 illustrates the cross-plot of various models developed in this study.

As evident from various error metrics and the cross-plot, the MLR-BS method did not demonstrate satisfactory performance for hydrogen solubility due to its lower complexity, with an R^2^ of 0.7682 and RMSE of 1.18E-01, generally underestimating solubility values. Among the presented methods, LSBoost-BS outperformed others, showing the best performance across the entire range of data used in this study with an R^2^ of 0.9997 and RMSE of 4.18E-03. ANN-BS also exhibited very good accuracy in predicting the output with an R^2^ of 0.9925 and RMSE of 2.13E-02, although it made errors in lower solubility values. In contrast to ANN-BS, RT-BS, while having high errors in average to high solubility values, showed very acceptable performance in lower values. The BT-BS method also, similar to RT-BS, provided reliable results for estimating lower solubilities. The GPR-BS method, like ANN-BS, was successful in estimating high solubilities. However, SVR-BS, unlike other methods, did not show good accuracy in predicting the low or high solubility range, but its output was reliable in average parameter values. CatBoost-BS and ET-BS methods also had very acceptable and close performance with R^2^ values of 0.9902 and 0.9901 respectively, and like ANN-BS and GPR-BS methods, they show very good performance in estimating medium and high solubility values. The application range of these methods based on the data obtained from cross-plots is summarized in Table 12.

**Table 12:** Accuracy of methods in each range of H_2_ solubility.

Range	ANN-BS	MLR-BS	RT-BS	SVR-BS	GPR-BS	BT-BS	LSBoost-BS	CatBoost-BS	ET-BS
Low solubility	✗	✗	✓	✗	✗	✓	✓	✗	✗
Medium solubility	✓	✗	✗	✓	✓	✗	✓	✓	✓
High solubility	✓	✗	✗	✗	✓	✗	✓	✓	✓

By checking the MAE values, it can be seen that according to this error criterion, the LSBoost-BS method with MAE = 2.80E-03 is the most accurate method, and after that, the ET-BS method with MAE = 9.78E-03 has a better performance compared to other methods. Based on this error criterion like other error factors, the MLR-BS method with MAE = 7.42E-02 is the least accurate method among the developed methods.

In general, regarding the accuracy of the developed models in this study, it can be stated that: LSBoost-BS > ANN-BS > CatBoost-BS > ET-BS > GPR-BS > BT-BS > RT-BS > SVR-BS > MLR-BS.

It is also noteworthy that, unlike other developed models, RT-BS and BT-bs methods did not calculate negative values as output due to their high accuracy in predicting low solubilities. This is in contrast to even the most accurate method identified here, LSBoost-BS, which sometimes yields negative values.

**Figure 11:**
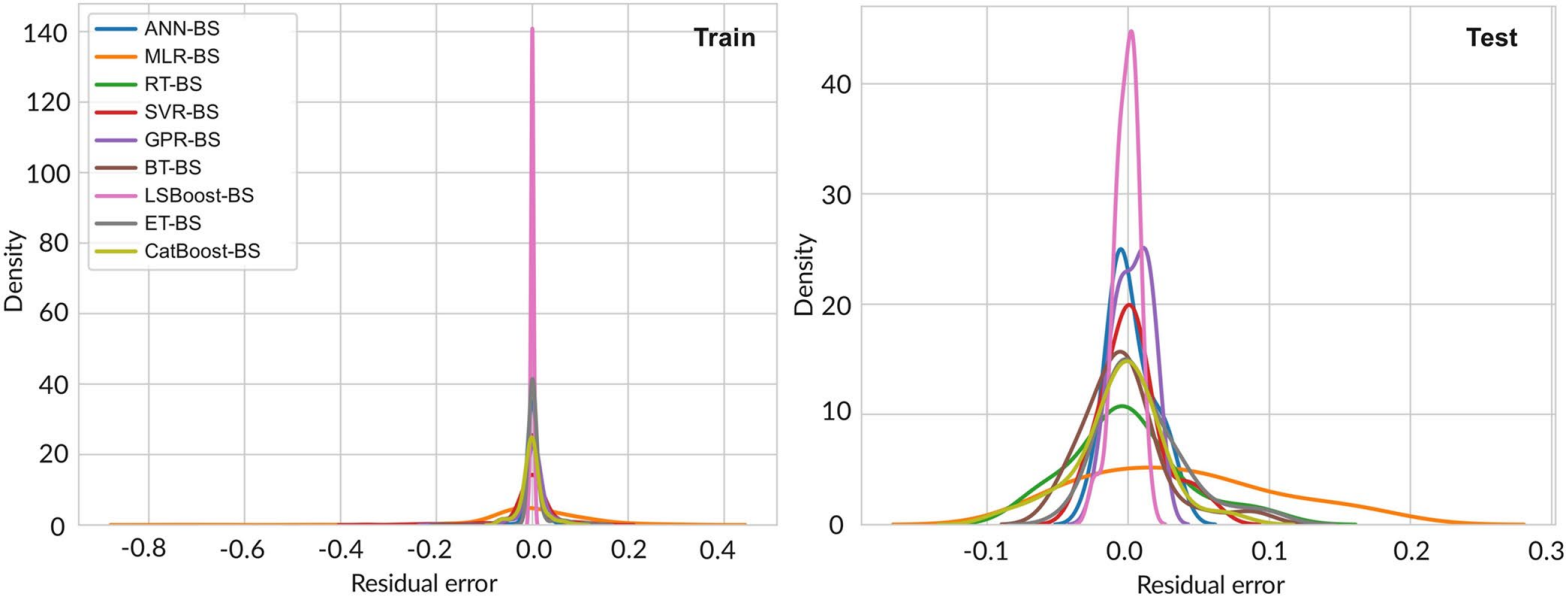
KDE plot of models for both train and test data.

A kernel density estimation (KDE) plot is shown in Fig. 11 for both train and test data to achieve a better evaluation of built models. Also in these plots, better performance of the LSBoost-BS method is obvious for train and test data. Also based on this plot except MLR-BS model other models show no bias.

**Figure 12:**
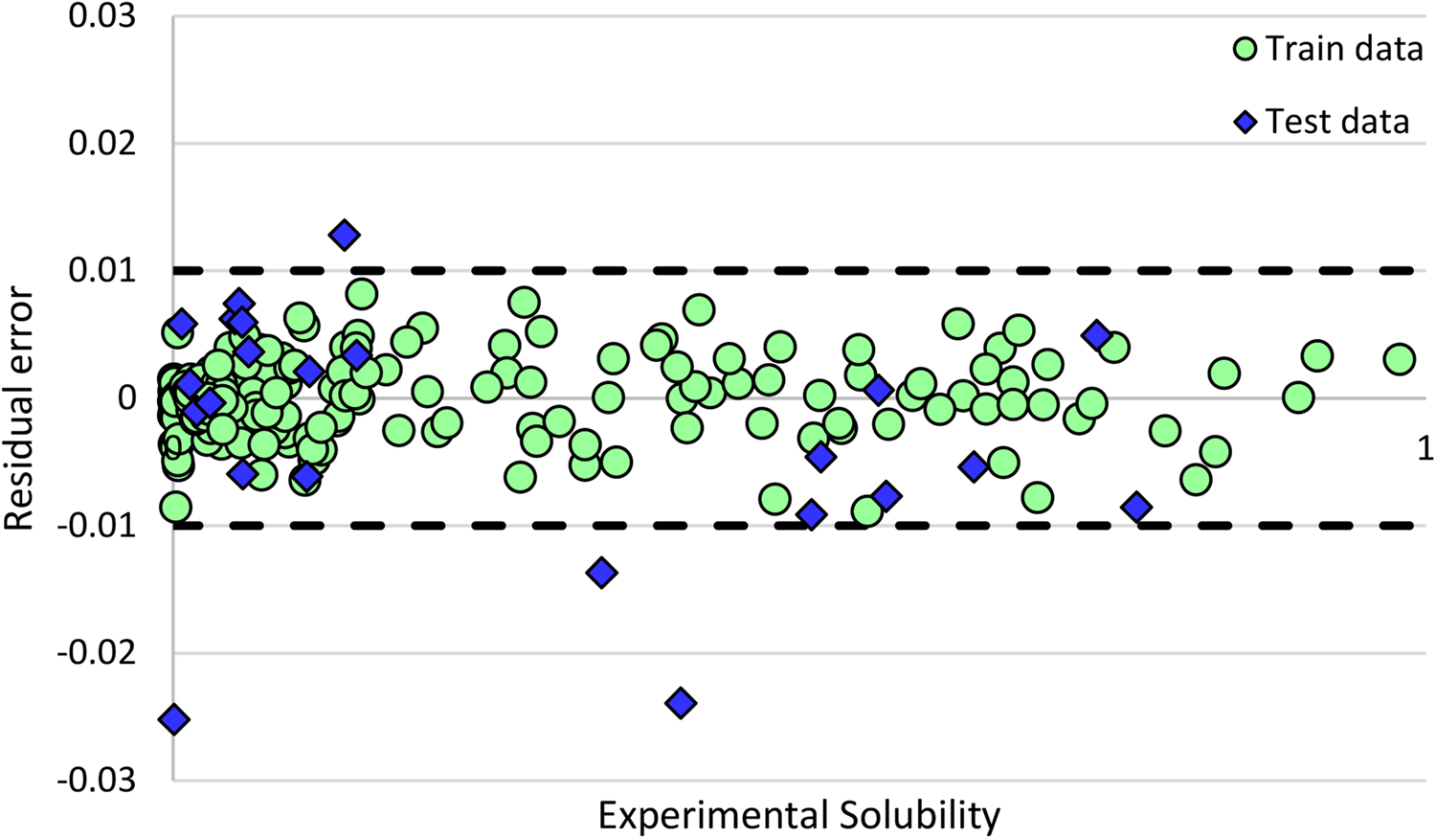
Residual error plot of LSBoost-BS model.

Following, for a more detailed examination of the selected method in this study, namely LSBoost-BS, a residual error plot of this model was generated. According to Fig. 12, the proposed method demonstrated excellent performance across the entire range of hydrogen solubility. The maximum residual error is -0.0252, and only 4 data points were estimated with an error greater than 0.01±. Among them, considering that the model error for 3 points is very low compared to the actual solubility values, these error values seem negligible.

**Figure 13:**
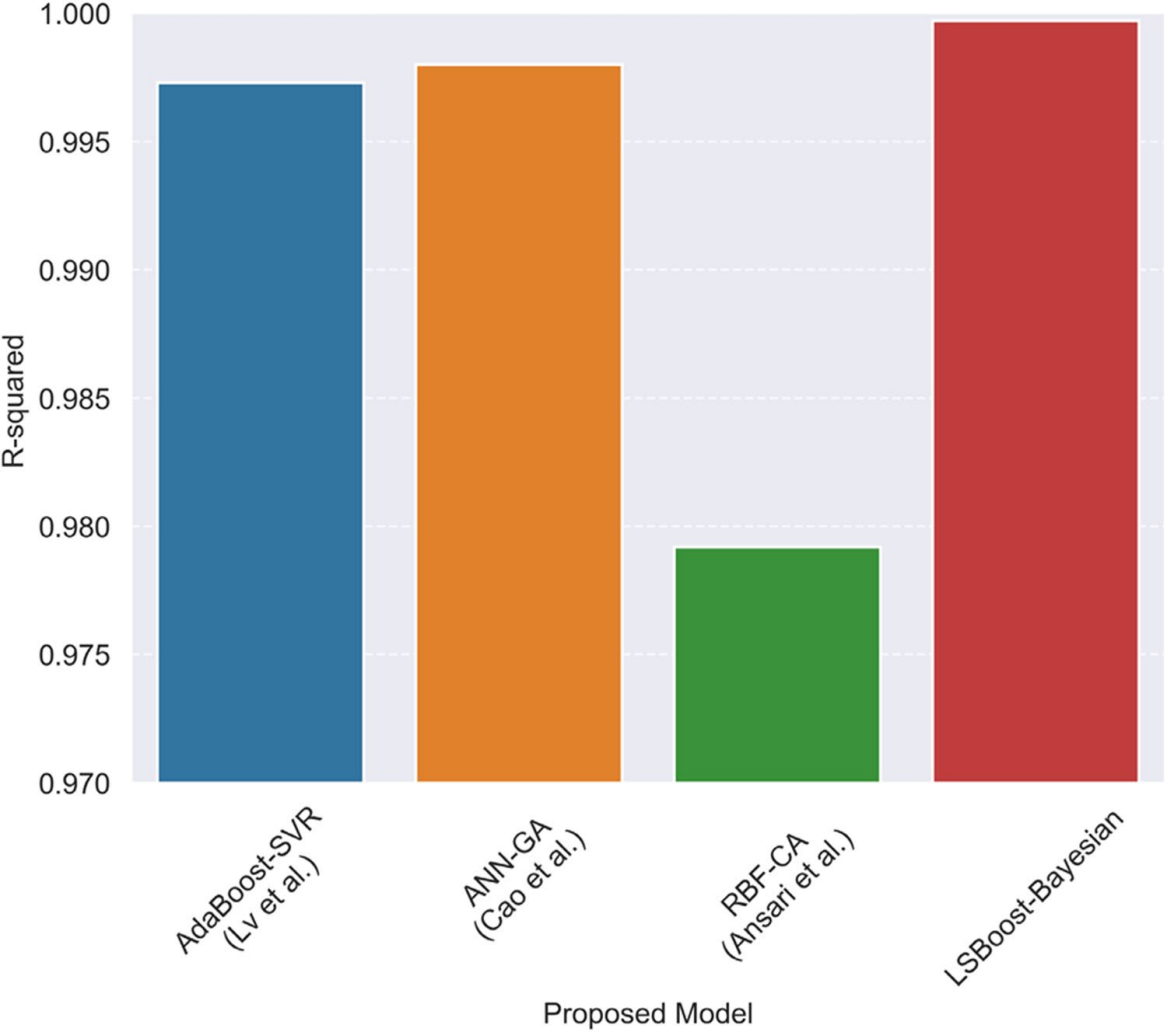
Accuracy of previously proposed models and proposed model in this study.

A comparison between the best methods used in previous studies and the proposed method in this research is illustrated in Fig. 13. It can be observed that although the proposed models in previous studies have shown very good accuracy in estimating hydrogen solubility, the proposed method has outperformed all previous studies in terms of performance.

### Sensitivity analysis

Sensitivity analysis to input data is considered a crucial step in the machine learning process. This analysis enhances our understanding of the sensitivity of machine learning algorithms to changes in input data and indicates whether their outputs are affected by these changes. It can contribute to a better understanding of the performance of machine learning algorithms and aid in selecting optimal parameters and configurations. Through this analysis, we can gain a better understanding of how algorithms behave in response to changes in input data and create better-performing models.

Following the determination of accuracy and the application of the proposed models, Pearson, Spearman, and Kendall correlation coefficients were calculated to assess the role of each parameter in estimating the output of each model.

Spearman’s correlation coefficient, also known as Spearman’s rank correlation coefficient, is a non-parametric measure of statistical dependence between two variables. This coefficient is used to assess how the relationship between two variables can be described using a monotonic function. A perfect Spearman correlation (+ 1 or -1) occurs when each variable is a monotonic function of the other. The Spearman coefficient, like any correlation calculation, is suitable for use with both continuous and discrete variables, including ordinal variables^136^.

Kendall’s rank correlation coefficient, often referred to as Kendall’s tau, is a common non-parametric statistic used to assess the statistical dependence between two measured variables. Kendall’s tau is applied to the ranked data observations, making it generally robust against outliers and tolerant of deviations from the assumption of normality in the data^137^.

The correlation values for various models and methods are presented in Table 13.

**Table 13:** Values of correlation between input parameters and predicted H_2_ solubility.

	Pearson			Spearman			Kendall		
	Pressure	Temperature	Salinity	Pressure	Temperature	Salinity	Pressure	Temperature	Salinity
ANN-BS	0.8199	0.0963	-0.5531	0.6939	0.0557	-0.7572	0.5916	0.0397	-0.5911
MLR-BS	0.9306	0.1090	-0.6321	0.8225	0.0949	-0.7743	0.6848	0.0631	-0.6574
RT-BS	0.8194	0.0763	-0.5558	0.6936	0.0131	-0.7267	0.6004	0.0083	-0.5746
SVR-BS	0.8685	0.1673	-0.5652	0.8130	0.1878	-0.7507	0.6894	0.1352	-0.6031
GPR-BS	0.8244	0.1009	-0.5540	0.7253	0.0699	-0.7716	0.6181	0.0525	-0.6195
BT-BS	0.8437	0.0924	-0.5511	0.7889	0.0632	-0.7248	0.6646	0.0461	-0.5725
LSBoost-BS	0.8188	0.1008	-0.5506	0.6797	0.0416	-0.7071	0.5769	0.0317	-0.5574
ET-BS	0.8195	0.1132	-0.5531	0.6153	0.0328	-0.7303	0.5101	0.0207	-0.5585
CatBoost-BS	0.8206	0.1098	-0.5358	0.6870	0.0749	-0.6961	0.5824	0.0550	-0.5432

It is evident that in all constructed models, temperature data had the least impact on the output. Additionally, pressure showed a direct correlation, while salinity exhibited a negative correlation. However, it is noteworthy that, unlike the Pearson and Kendall coefficients, in ANN-BS, RT-BS, GPR-BS, LSBoost-BS, ET-BS, and CatBoost-BS models, the Spearman coefficient indicates that temperature has the most significant impact on the model output, albeit a very slight difference. In general, by averaging the parameter influences across various methods, it can be concluded that pressure has more effect on the hydrogen solubility in water.

**Figure 14:**
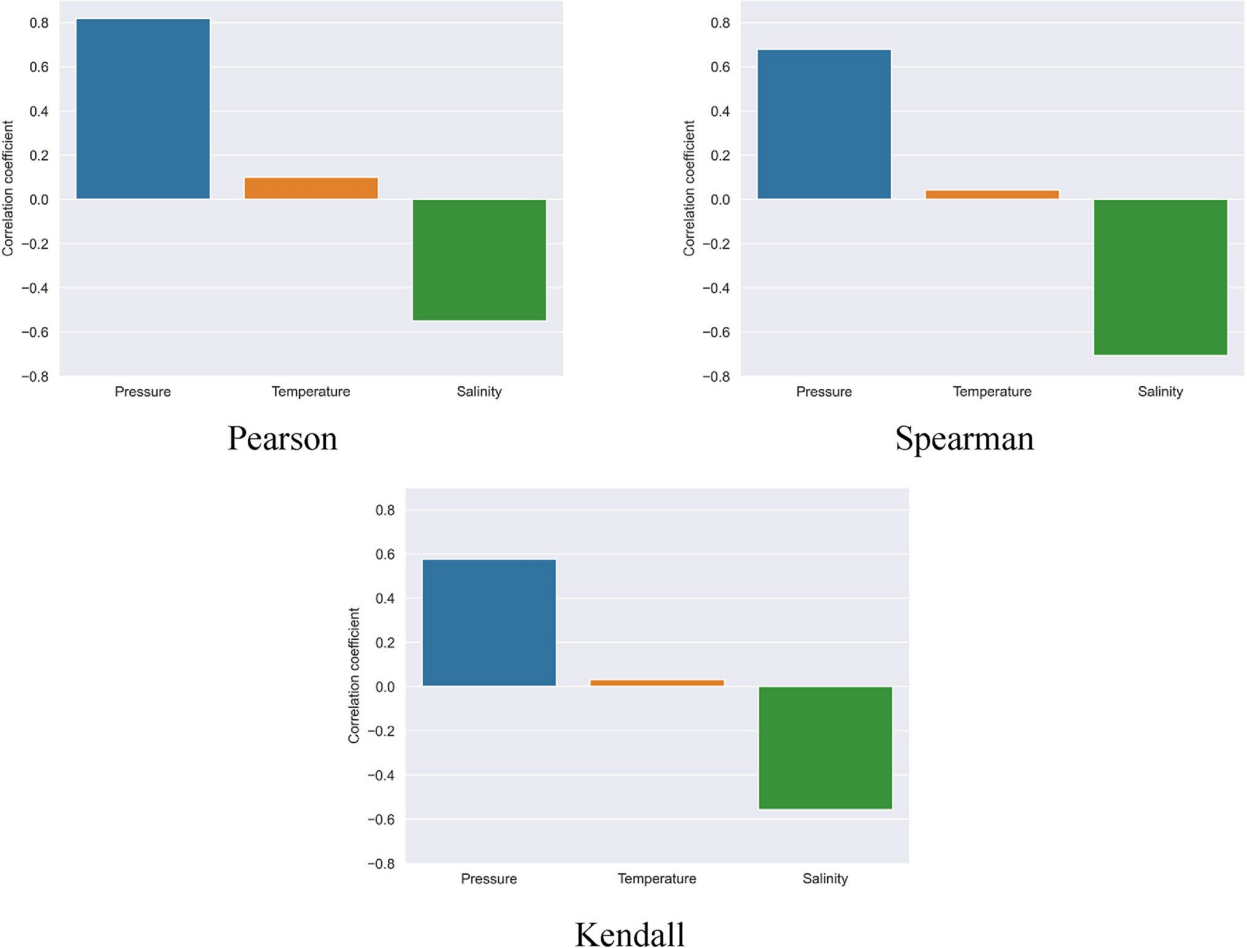
Correlation coefficients of input parameters calculated for LSBoost-BS model.

Figure 14 illustrates different correlation coefficients for the best model of this study, LSBoost-BS.

As can be seen, based on PCC values, among the developed models, the MLR-BS model is most affected by the pressure parameter, while the superior model, LSBoost-BS, is the least affected by this parameter compared to the rest of the models. In general, regarding the effect of pressure on the output of different models, it can be said MLR-BS > SVR-BS > BT-BS > GPR-BS > CatBoost-BS > RT-BS > ANN-BS > ET-BS > LSBoost-BS.

Concerning the temperature parameter, the least impact is related to the RT-BS model, and the most impact is related to the SVR-BS model and in general SVR-BS > ET-BS > MLR-BS > GPR-BS > BT-BS > ANN-BS > CatBoost-BS > LSBoost-BS > RT-BS.

Also, like pressure, the highest and lowest effectiveness of the salinity parameter is related to the MLR-BS and LSBoost-BS models, respectively, and in general, it can be said: MLR-BS > GPR-BS > ET-BS > SVR-BS > ANN-BS > RT-BS > BT-BS > LSBoost-BS > CatBoost-BS regarding the effectiveness of different models of this parameter.

The important point is the low effectiveness of each of the parameters separately in the LSBoost model compared to the rest of the methods.

### Implementation for H_2_ storage in saline aquifers

Utilizing machine learning techniques for estimating hydrogen solubility in aqueous solutions presents a promising avenue for advancing hydrogen storage in deep saline aquifers. The implementation of such methodologies holds the potential to revolutionize the landscape of energy storage by enhancing storage capacity, mitigating environmental damage, and ensuring cost efficiency.

One significant advantage lies in the enhanced storage capacity enabled by leveraging machine learning algorithms. These techniques allow for a more precise prediction of hydrogen solubility in saline aquifers, enabling the optimization of storage conditions for maximal capacity utilization. By accurately estimating solubility, operators can determine the optimal injection parameters, such as pressure and temperature, to maximize the volume of hydrogen stored in the aquifer while ensuring safe and efficient operations.

Moreover, this approach offers the potential for significantly reduced environmental damage compared to traditional storage methods. By utilizing deep saline aquifers for hydrogen storage, the risk of leakage or contamination of groundwater sources is minimized. Machine learning algorithms can aid in identifying suitable aquifers with minimal risk of adverse environmental impacts, thereby ensuring the integrity of both the storage facility and surrounding ecosystems. Additionally, the utilization of saline aquifers for hydrogen storage can contribute to the reduction of greenhouse gas emissions, as hydrogen produced from renewable sources can be stored underground, effectively serving as a form of carbon-free energy storage.

Furthermore, the implementation of machine learning techniques for hydrogen storage in saline aquifers can offer substantial cost efficiencies. By accurately predicting solubility and optimizing storage conditions, operators can minimize the need for costly infrastructure modifications or extensive monitoring systems. Additionally, the use of saline aquifers, which are abundant and often underutilized geological formations, can provide a cost-effective solution for long-term hydrogen storage. Overall, the implementation of machine learning for hydrogen storage in saline aquifers represents a promising approach that combines enhanced storage capacity, environmental sustainability, and cost efficiency, paving the way for a more sustainable energy future.

### Limitations

This study has a few key limitations that should be noted. One primary limitation is the relatively low number of data points (226) used to train and test the machine learning models. The limited data size may restrict the model’s ability to generalize across broader scenarios or unseen data, potentially affecting the reliability of the predictions in real-world applications.

Another limitation is the exclusive use of Bayesian optimization for hyperparameter tuning. While this method is effective, relying on a single optimization technique may reduce the potential exploration of other optimization approaches, which could lead to alternative or improved model performance. This lack of diversity in optimization methods might limit the flexibility of the models.

Additionally, the applicability of the developed models is confined to the specific data range outlined in the methodology section. The models were trained and validated using data within defined ranges of temperature, pressure, and salinity, and their performance outside these boundaries is uncertain. As a result, the models may not perform reliably when applied to conditions that fall outside the limits of the data used in this study.

### Future works

In this research, the solubility of hydrogen in aqueous environments was investigated using a variety of machine learning techniques. Nine methods were employed, including MLR-BS, ANN-BS, SVR-BS, RT-BS, GPR-BS, BT-BS, LSBoost-BS, ET-BS, and CatBoost-BS. Utilizing a dataset of 226 data points gathered from previous studies, we optimized the hyperparameters of these models using Bayesian optimization.

The results revealed that LSBoost exhibited the best performance among the developed models. Although the collected data encompass a wide range of parameter values, further data collection could enhance the development of more comprehensive models.

To guide future research efforts, several suggestions are proposed:

- Utilize larger databases of hydrogen solubility in water to construct more comprehensive models.
- Incorporate hydrogen solubility data from various environments to build unified and powerful models.
- Optimize hyperparameters of different models using alternative methods such as PSO, GA, etc.
- Explore newer machine learning methodologies that may outperform the developed approaches.

## Conclusions

In this research, seven machine learning models were developed for estimating hydrogen solubility in aqueous solutions. Initially, data from previous studies were collected. To ensure sufficient data dispersion, scatter plots of input parameters against each other and the output were created. The linear dependence of each parameter on the others was then calculated. In the next step, data larger or smaller than 3 standard deviations from the mean were removed to improve the quality of the data used in model development. After developing the models, error function methods and graphical methods were employed to evaluate the performance of the data. It was determined that the LSBoost method with an R² of 0.9997 exhibited the best performance in estimating hydrogen solubility. Generally, the performance of the methods was in the order of LSBoost-BS > ANN-BS > CatBoost-BS > ET-BS > GPR-BS > BT-BS > RT-BS > SVR-BS > MLR-BS. Subsequently, to evaluate the performance range of the superior model, a residual error plot against hydrogen solubility was drawn, indicating that this model performs very well for almost all solubility ranges. Also, to investigate the effect of each input parameter on the model’s estimated output, three correlation calculation methods were used. The least effect on the output among the parameters belonged to temperature, while pressure showed a positive correlation, and salinity exhibited a negative correlation with hydrogen solubility. Considering the ease of using the developed model, its application alongside other methods such as state equations can contribute to improving the efficiency of hydrogen storage operations in practical scenarios.

This study offers several advantages, particularly the use of a wide range of machine learning models, which enhances the robustness of the predictions by allowing for comprehensive evaluation across different approaches. Additionally, the removal of outliers before constructing the models ensures higher data quality and more reliable results. The study also utilizes multiple evaluation methods, including various error metrics, graphical assessments, and kernel density estimation plots, providing a thorough analysis of model performance. On the other hand, the relatively low number of data points may limit the model’s ability to generalize across broader scenarios.

Given the high accuracy of the developed model (LSBoost-BS) and its fast estimation speed in predicting hydrogen solubility, integrating it alongside other methods such as state equations can enhance the efficiency of hydrogen storage in brackish aquifers. This integration can lead to increased storage efficiency, reduced environmental risks, and lower costs. Additionally, leveraging the reliable performance of this model in future studies and on larger databases will aid in constructing even more robust models.

## Nomenclature

ANN Artificial neural network

BS Bayesian algorithm

BT Bagged trees

CatBoost Categorical Boosting

ET Extra trees

SVR Support vector regression

RT Regression tree

GPR Gaussian process regression

LSBoost Least-square boosting

MAE Mean absolute error

MLR Multiple linear regression

MSE Mean square error

RMSE Root mean square error

RBF Radial basis function

CA Cultural algorithm

PCC Pearson correlation coefficient

## Data Availability

All data generated or analyzed during this study are included in this published article.
